# Water Stress Inhibits Germination While Maintaining Embryo Viability of Subtropical Wetland Seeds: A Functional Approach With Phylogenetic Contrasts

**DOI:** 10.3389/fpls.2022.906771

**Published:** 2022-05-31

**Authors:** Arvind Bhatt, L. Felipe Daibes, David J. Gallacher, Alfredo Jarma-Orozco, Marcelo F. Pompelli

**Affiliations:** ^1^Lushan Botanical Garden, Chinese Academy of Sciences, Jiujiang, China; ^2^Departamento de Biodiversidade, Instituto de Biociências, Universidade Estadual Paulista, Rio Claro, Brazil; ^3^Northern Western Australia and Northern Territory Drought Hub, Charles Darwin University, Sydney, NT, Australia; ^4^Grupo Regional de Investigación Participativa de los Pequeños Productores de la Costa Atlantica, Universidad de Córdoba, Montería, Colombia

**Keywords:** drought, germination, hydrotime, PEG6000, osmotic potential, subtropical forest

## Abstract

Wetland species commonly exhibit a range of strategies to cope with water stress, either through drought tolerance or through avoidance of the period of limited water availability. Natural populations provide a genetic resource for ecological remediation and may also have direct economic value. We investigated the effects of drought stress on the seed germination of wetland species. Nineteen species were germinated in four concentrations of polyethylene glycol 6000 (PEG) and were evaluated daily (12-h light photoperiod) or after 35 days (continuous darkness) to determine seed germination under water stress. Germination percentage decreased with an increase in polyethylene glycol 6000 (PEG) concentration, but species’ germination response to PEG concentration varied significantly. Seeds recovered their germinability after the alleviation of water stress, but the extent of recovery was species-dependent.

## Introduction

The incidence and intensity of drought and temperature extremes are increasing with climate change and are a serious threat to many natural ecosystems (Smith, 2011; Cardinale et al., 2012; Čanak et al., 2020) due to the implications for germination and seedling survival (Walck et al., 2011; Fernández-Pascual et al., 2019). Environmental factors such as temperature, light, and availability of moisture are the most important factors regulating seed dormancy and germination (Baskin and Baskin, 2014). Moisture availability is fundamental for seeds to initiate water absorption (Kestring et al., 2009), and osmotic solutions can be applied to evaluate germination patterns, simulating drought stress under low water potential (Michel and Kaufmann, 1973; Bradford et al., 2013). Drought stress may negatively impact plant regeneration, growth, and survival (Du et al., 2019; Ding et al., 2020) due to the reduced osmotic potential of dehydrated seeds restricting their metabolism (Bradford et al., 2013). Water is the main abiotic factor limiting seed germination and early seedling growth (Ansari et al., 2013; Bhatt et al., 2020a). Effects of severe drought stress vary with species, and different taxa may display different adaptation strategies to survive under such stressful events (Volis and Bohrer, 2013; Baskin and Baskin, 2014; Miranda et al., 2014; Bhatt et al., 2019). Evaluating drought tolerance during seed germination could assist with understanding population persistence and community assembly patterns, and how these may be affected by a changing climate.

Ecological functions such as dispersal mode, germination, seedling establishment, growth rate and plant size, competition, and survival can be predicted by easily obtained seed traits such as seed mass and shape (Moles and Westoby, 2004; Moles et al., 2005; Jiménez-Alfaro et al., 2016; Saatkamp et al., 2019). Seed mass is positively correlated with higher germination percentages and seedling establishment under drought stress, due to higher levels of food reserves (Kos and Poschlod, 2008; Čanak et al., 2020; Koirala and Neff, 2020).

Lower drought tolerance during germination is a cautious strategy of reduced fitness when conditions are less suitable and is likely to correlate negatively with seed size (Kos and Poschlod, 2008). The relationships between germination response to drought stress and seed size could be important to understand functional regeneration strategies. Phylogenetic constraints also influence seed trait patterns at the community level and thus inform the ecological roles of functional traits (Bu et al., 2008; Wang et al., 2009).

Species differ in their life span (annual vs. perennial) and strategy toward drought (tolerance vs. avoidance). Annual species usually show stress-escaping strategies due to presenting rapid phenological development and a high degree of plasticity, being able to complete their life cycle before water deficit becomes severe enough to cause physiological damage (Kooyers, 2015). Perennial species avoid water stress both by absorbing water through an abundant root system and by reducing transpiration through stomatal closure or laminar morphology. Drought tolerance is a mechanism through which a plant maintains metabolism even with the reduction of tissue water potential, mainly due to the accumulation of compatible solutes or osmolytes, osmoprotective proteins, and antioxidant capacity (Verslues et al., 2006). Poorter et al. (2012) described perennial species as drought-tolerant and able to allocate more resources to roots, thus improving their ability to source moisture (Roumet et al., 2006). However, few studies have compared germination response to drought between annual and perennial species from subtropical wetlands. We expect differences in life span to reflect the variation in drought tolerance during the germination stage.

In wetland areas, species commonly show a range of strategies to cope with osmotic stress (e.g., salinity) or dry spells, either by being drought tolerant or by escaping drought during the period of limited water availability (Capon, 2003). It has been predicted that drought frequency will increase in the near future (Zhang et al., 2020), which may cause stress to wetland areas causing the extinction of some species. The climate of south China is relatively humid when compared with the northern part of the country (Huang et al., 2017), but drought incidents are increasing in frequency and severity in southern China including Jiangxi Province in recent years (Ding et al., 2020; Zhang et al., 2020). Poyang lake, one of the largest freshwater lakes located in Jiangxi Province, China, is becoming subjected to seasonal water level fluctuation, which is severely impacting wetland areas (Feng et al., 2016; Mei et al., 2016). The gradual degradation of wetland areas will alter the community composition of wetland plants by disrupting and segregating their habitats (Feng et al., 2016).

The impacts of drought on species from subtropical monsoonal climatic conditions of wetland areas around the Poyang lake remain currently unexplored. In this study, we aimed to investigate: (i) the ability of seeds from 19 different species to germinate under a gradient of water potentials, (ii) germination recovery after the alleviation of drought stress, and (iii) the relationship of seed mass/size, life span, and phylogeny with drought tolerance. The results of this study will help to explain the interspecific variability in drought tolerance strategies among wetland species, aiding to predict the consequences of drought on species composition and performance in a changing world.

## Materials and Methods

### Seed Collection and Storage

Freshly mature seeds were collected at the time of natural seed dispersal (between May and November) from different areas of Jiujiang, China, during 2020 (Table 1). The climate of Jiujiang is categorized as subtropical monsoon, where winter (December–February) is cold with a min/max of 3–11°C, while summer (July–August) is hot with a maximum temperature of 39°C. Rainfall events occur throughout the year, but precipitation is greatest during the monsoon (May–July).

**TABLE 1:** Location and other details of species.

Species	Code	Family	Collection time	Place	Latitude	Longitude	Altitude (msl)	Habit	Habitat	Wetland species +	Occasionally occur in wetland ^x^
*Aeschynomene indica* L.	AI2	Fabaceae	September	Minshan	29°41’27.62″	116°4’16.52″	2.41	Annual	Open area	+	
*Beckmannia syzigachne* (Steud.) Fernald	BS2	Poaceae	May	Minshan	29°29’47.28″	115°53’14.85″	116.37	Annual	Open area	+	
*Bidens pilosa* L.	BP	Asteraceae	November	Gutang	29°40’13.03″	116°5’33.82″	13.55	Annual	Open area		^x^
*Echinochloa crus-galli* (L.) P.Beauv. *	EC2	Poaceae	September	Gutang	29°40’10.99″	116°5’33.52″	16.24	Annual	Open area	+	
*Echinochloa crus-galli var. mitis (Pursh) Peterm.*	ECM	Poaceae	November	Gutang	29°40’03.72″	116°6’33.32″	24.54	Annual	Open area	+	
*Eclipta prostrata* (L.) L.	EP	Asteraceae	October	Gaolong	29°35’44.86″	116°3’38.96″	62.06	Annual	Open area	+	
*Juncus effusus* L.*	JE2	Juncaceae	June	Saiyang	29°32’18.53″	115°53’25.77″	107.14	Perennial	Streamside	+	
*Juncus prismatocarpus* R.Br.	JP	Juncaceae	June	Yujiahe	29°41’59.66″	116°3’46.58″	8.02	Perennial	Waterlogged area	+	
*Kyllinga brevifolia* Rottb.*	KB	Cyperaceae	September	Gutang	29°40’31.937″	116°6’16.88″	−0.04	Perennial	Open area	+	
*Leptochloa chinensis* (L.) Nees *	LC2	Poaceae	November	Gutang	29°39’25.56″	116°6’22.49″	25.76	Annual	Open area	+	
*Leptochloa panicea* (Retz.) Ohwi *	LP	Poaceae	September	Gutang	29°40’27.89″	116°5’38.68″	−7.28	Annual	Streamside	+	
*Lophatherum gracile* Brongn.	LG	Poaceae	November	Guling	29°33’12.98″	115°57’56.01″	947.81	Perennial	Forests		^x^
*Melochia corchorifolia* L.	MC	Sterculiaceae	October	Minshan	29°29’31.03″	115°53’06.26″	98.94	Perennial	Open area	+	
*Polypogon fugax* Nees ex Steud.	PF	Poaceae	May	Weijia	29°40’31.39″	116°5’29.14″	4.94	Annual	Streamside		^x^
*Polygonum lapathifolium* var. salicifolium Sibth.	PLS2	Polygonaceae	July	Gutang	29°40’13.03″	116°5’33.82″	13.55	Annual	Grassland	+	
*Rorippa globosa* (Turcz. ex Fisch. & C.A. Mey.) Hayek	RG2	Brassicaceae	June	Yujiahe	29°41’33.94″	116°2’52.59″	13.29	Annual	Open area	+	
*Rumex japonicus* Houtt.	RJ	Polygonaceae	June	Yujiahe	29°40’55.23″	116°4’21.73″	8.95	Perennial	Cultivated area	+	
*Sporobolus fertilis* (Steud.) Clayton*	SPF2	Poaceae	November	Lianhua	29°37’14.35″	115°58’33.58″	216.52	Perennial	Open area		^x^
*Veronica persica* Poir.	VP	Scrophulariaceae	June	Yujiahe	29°41’02.48″	116°4’28.78″	7.19	Annual	Open area		^x^

*C4 species (Zhai Z K, Zhao M, Wei Y S, Liu H Q, Bai Y, 2020. The List of C4 Plant in China. Shaanxi Forest Science and Technology,48(05): 7189-7193).

***+**Wetland species are defined as per the Convention on Wetland of International Importance, especially as Waterfowl habitat (1971).*

*^x^eflora of China.*

We categorized the study species into two groups: (i) wetland species (defined as per Convention on Wetlands of International Importance, especially as Waterfowl Habitat -- 1971) and (ii) species that may be found in wetland habitats but are not limited to them.^1^ Seeds of each species were collected from more than 35 individuals spaced at least 2 m apart, to ensure the genetic diversity of the population. Seeds were cleaned and dried to 5–8% moisture content (optimal conditions for most of the orthodox seeds) using 15°C and 20% relative humidity before storing at −18°C at the Lushan Botanical Garden, China, until the experiment started in September 2021. Before starting the germination experiments, seeds were retrieved from −18°C storage and allowed to equilibrate to room temperature for 24 h.

### Seed Morphology

Fresh seed mass was determined for each study species by weighing three 100-seed replicates using an analytical balance (Sartorius Analytical Balance mod. ENTRIS224-1S, Bradford, MA, United States; accurate to 0.1 mg). Seed length, width, and breadth were measured on 15 seeds per species using a Stereo Microscope (Nikon SMZ800N; Nikon Instruments Inc. Melville, NY, United States) fitted with a camera IMG-SC600C (iMG Biotechnology Co., Ltd, Suzhou, Jiangsu, China). Seed shape index was calculated as described in Thompson et al. (1993).

### Effects of Water Stress on Seed Germination

Effect of drought stress (i.e., low water potential) on germination was determined using polyethylene glycol-6000 (PEG 6000, Merck Group, Darmstadt, Germany, part number 8170075000). The GerminaR package version 2.1.3 (Lozano-Isla et al., 2019) was used to calculate the fresh weight of PEG 6000 to Ψ_w_ = 0, −0.3, −0.6, and −0.9 MPa. These osmotic potentials were used based on our preliminary study. Seeds were incubated in 9-cm-diameter Petri dishes on one sheet of Whatman No. 1 filter paper moistened with distilled water (control) or one of the three concentrations of PEG. To prevent evaporation, Petri dishes were sealed with parafilm. Four replicates of 25 seeds each were used for each treatment and placed in a germinator set at 12/12 h cycles of 20/30°C in light conditions (12-h light photoperiod) and also under constant darkness (dark treatment). Petri dishes subjected to the dark treatment were wrapped in aluminum foil. The temperature used to incubate the seeds was chosen because it was found to be optimal for the germination of these species (data not shown). The higher temperature period coincided with the light cycle in the 12-h light photoperiod. Germinated seeds were counted daily and removed from light treatments for 35 days after seed soaking; but in the dark treatment, they were counted only at day 35. Germination was defined as the emergence of a radicle >2 mm through the external integument, as proposed by the International Seed Testing Association (Allen and Alvarez, 2020). Thereafter, germination percentage (G%), mean germination time (MGT), and synchrony (SYN) were computed in 12-h light photoperiod seeds using GerminaR (Lozano-Isla et al., 2019) in accordance with formulas expressed in Bhatt et al. (2020a). The photoblastism was assessed by calculating the relative light germination index (RLG) as described in Milberg et al. (2000) and Flores et al. (2011).

### Germination Recovery

Non-germinated seeds from the previous water potential treatments with light exposure were tested for germination in conditions free from water stress. The remaining seeds were rinsed four times with distilled water, placed in newly prepared Petri dishes, as described previously, and moistened with distilled water. These Petri dishes were placed in light at 20/30°C, and germinated seeds were counted daily for 25 days to test their ability to recover germination. At the end of the experiment, a cut test with scalpel to evaluate the embryo status (living and therefore white and turgid, or brown and therefore dead) under a binocular microscope was carried out to evaluate the viability of ungerminated seeds (data not shown).

### Germination Traits Grouping and Phylogenetic Analysis

To group the seeds by similar germination behavior, we selected the following traits: germination at 0 MPa under white light (control), to detect primary dormancy; effect size of −0.3 MPa in relation to control, to detect drought tolerance; the RLG, to detect the light sensitivity; and germination from recovery after −0.9 MPa, to detect resistance to rehydration. The groups were established via K-means and then plotted in a cluster plot. Those germination behavior traits and seed size for each species were used to obtain scored values from principal component analysis.

For detection of a phylogenetic trace on seed germination behavior and seed size, we built a phylogenetic tree with the “V.PhyloMaker” package (Jin and Hong, 2019). Then, the phylogenetic signal was analyzed via the Pangel’s λ, which is based on the Brownian motion evolution model (“phytools”).

### Data Analysis

The influence of incubation temperature (IT) on three dependent variables (i.e., germination percentage, mean germination time, and synchrony) was performed using GerminaR software (Lozano-Isla et al., 2019). All data were analyzed by ANOVA, and means were compared using an SNK test (*P* < 0.05) by Statistic version 14.0 (StatSoft, Tulsa, OK, United States). Correlations among variables were assessed using Pearson’s correlations using Sigmaplot version 14.0 (Systat Software Inc., San Jose, CA, United States).

## Results

### Characterization of Study Species

The 19 study species are listed in Table 1. To facilitate some analyses, species’ names were replaced by a code representing each species. Of the 19 species, eight belonged to the Poaceae family; two to each of Asteraceae, Juncaceae, and Polygonaceae; and one from each of Fabaceae, Cyperaceae, Sterculiaceae, Brassicaceae, and Scrophulariaceae. Twelve species were annuals and seven perennials. Most of the study species (13) inhabit open areas, while three are riparian and the remainder are from cultivated lands (*Rumex japonicus*), forests (*Lophatherum gracile*), or waterlogged areas (*Juncus prismatocarpus*). In total, 14 species were classified as wetland species.

Seed dimensions varied strongly among the study species, with two of them (*Leptochloa chinensis* and *Kyllinga brevifolia*) having a seed length of 838.74 ± 15.32 and 402.16 ± 12.68 μm, respectively (Table 2). For the other 17 species, seed length varied from 0.48 ± 0.01 μm (in *Juncus effusus*) to 9.16 ± 0.33 μm in *Bidens pilosa* and *Eclipta prostrata*, whose seeds were 19.1-fold longer than *J. effusus*. Comparing the extremes, seeds of *L. chinensis* (the largest) had an increased ratio up to 1,747-fold longer than *J. effusus* seeds (the smallest). A similar pattern was verified regarding seed width, where *L. chinensis* and *K. brevifolia* presented values of 457.34 ± 14.24 and 225.28 ± 5.67 μm, respectively (Table 2). If we consider the two extremes, *L. chinensis* and *J. prismatocarpus* (0.25 ± 0.01 μm), *L. chinensis* seeds are 1,829.4-fold wider than *J. prismatocarpus*. Seed shape index had less variation comparing the rounder seeds, with values close to 0 (i.e., 0.02 ± 0.01; *R. japonicus*), and the ones tending to be more flattened/elongated, with higher values (0.19 ± 0.01; *B. pilosa*; Table 2). Such variation was thus limited to D_shape_ = 0.17 in seed shape index in favor of *B. pilosa* seeds. In contrast, the fresh mass of 100 seeds varied greatly between the lightest seeds (*L. chinensis*; 0.04 ± 0.01 mg) and the heaviest ones (*A. indica*; 1,004.00 ± 8.33). Seeds of *Aeschynomene indica* were 25,100-fold heavier than *L. chinensis*. Other features, such as seed morphology and seed color, are also shown in Table 2.

**TABLE 2:** Seed length (SL), seed width (SW), seed height (SH), seed shape (SS), fresh weight of 100 seeds (P100), seed morphology, and seed color measured in 19 plant species.

Plant species	SL (μm)	SW (μm)	SS	P100 (mg)	Seed morphology	Color
*A. indica*	3.49 ± 0.04 e	2.30 ± 0.03	0.04 ± 0.01 i	1,004.00 ± 8.33 a	Reniform	Blackish brown
*B. syzigachne*	1.72 ± 0.02 i	0.57 ± 0.01	0.11 ± 0.01 c	33.33 ± 1.33 h	Long elliptic	Tawny
*B. pilosa*	9.16 ± 0.33 c	0.73 ± 0.02	0.19 ± 0.01 a	149.33 ± 6.67 f	Cylindrical	Dark brown
*E. crus-galli*	2.59 ± 0.10 g	1.48 ± 0.02	0.06 ± 0.01 fg	107.67 ± 7.42 g	Oval	Brown
*E. crus-galli var. mitis*	2.95 ± 0.04 f	1.61 ± 0.02	0.07 ± 0.01 f	201.33 ± 4.81 e	Oval	Brown
*E. prostrata*	9.16 ± 0.33 c	0.73 ± 0.02	0.19 ± 0.01 a	28.00 ± 2.31 hi	Oval	Light brown
*J. effusus*	0.48 ± 0.01 l	0.28 ± 0.01	0.06 ± 0.01 fg	4.00 ± 0.01 i	Oval-shaped Oblong	Tawny
*J. prismatocarpus*	0.55 ± 0.01 l	0.25 ± 0.01	0.08 ± 0.01 e	4.00 ± 0.02 i	Long oval	Wax yellow
*K. brevifolia*	402.16 ± 12.68 b	225.28 ± 5.67	0.09 ± 0.01 d	8.00 ± 0.01 i	Obovate oblong	Brown
*L. chinensis*	838.74 ± 15.32 a	457.34 ± 14.24	0.08 ± 0.01 e	0.04 ± 0.01 i	Oblong sphere	Brown
*L. panicea*	0.60 ± 0.01 l	0.41 ± 0.01	0.04 ± 0.01 j	4.00 ± 0.01 e	Oblong sphere	Brown
*L. gracile*	3.94 ± 0.11 d	1.06 ± 0.02	0.12 ± 0.01 b	306.67 ± 4.81 c	Long oval	Brown
*M. corchorifolia*	2.10 ± 0.02 h	1.50 ± 0.03	0.03 ± 0.01 j	229.33 ± 2.67 d	Ovoid	Brown-black
*P. fugax*	0.93 ± 0.01 k	0.41 ± 0.01	0.09 ± 0.01 d	5.33 ± 1.33 i	Oval	Brown
*P. lapathifolium*	2.10 ± 0.03 h	1.63 ± 0.03	0.12 ± 0.01 bc	426.67 ± 23.25 b	Broad-ovate	Brown/Fulvous
*R. globosa*	0.56 ± 0.02 l	0.47 ± 0.02	0.09 ± 0.01 d	5.33 ± 1.33 f	Broadly ovate	Light brown
*R. japonicus*	2.21 ± 0.04 h	1.55 ± 0.04	0.02 ± 0.01 j	142.67 ± 7.42 c	Broad-ovate	Reddish/Dark-brown
*S. fertilis*	1.03 ± 0.02 k	0.75 ± 0.01	0.05 ± 0.01 gh	25.33 ± 1.33 hi	Oblong oval	Brown
*V. persica*	1.59 ± 0.03 g	1.21 ± 0.04	0.05 ± 0.01 h	49.33 ± 2.67 h	Oblong	Brown

*In each column, different lowercase letter denotes statistical significance at P ≤ 0.05.*

### Effects of Osmotic Stress on Seed Germination

**FIGURE 1:**
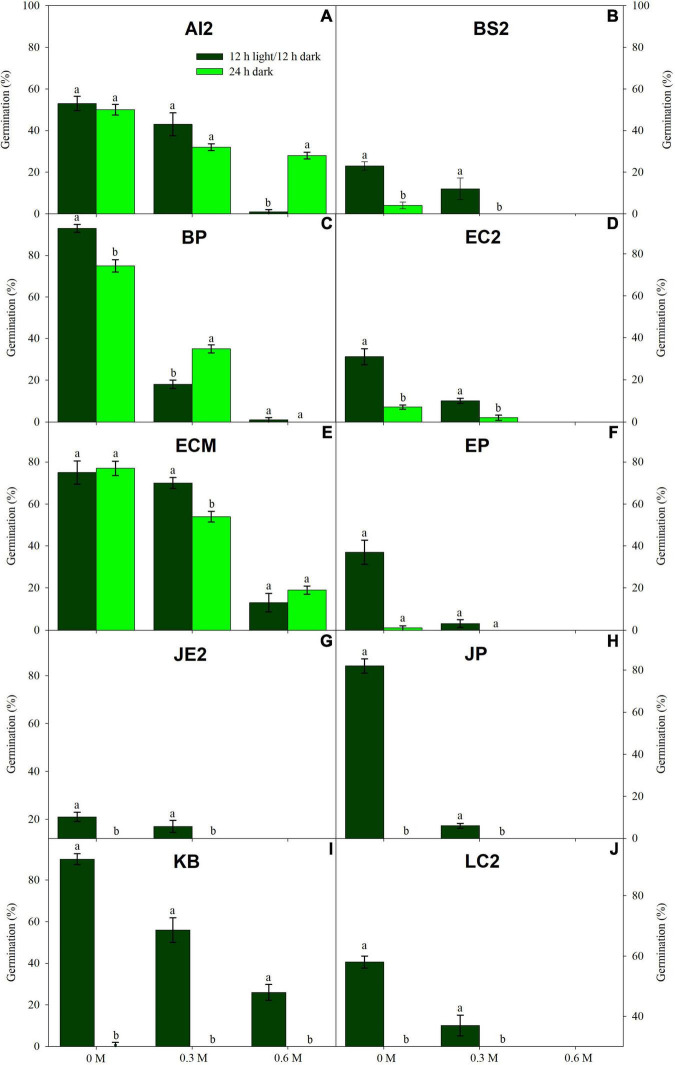
Germination in light (dark green) and dark treatments (light green) under three PEG6000 concentrations for 10 wetland species occurring in Jiujiang, China. Vertical bars denote ± SE around the mean. Within features, different lowercase letters indicate significance at *P* ≤ 0.05. For more details on each study species, see Table 1.

**FIGURE 2:**
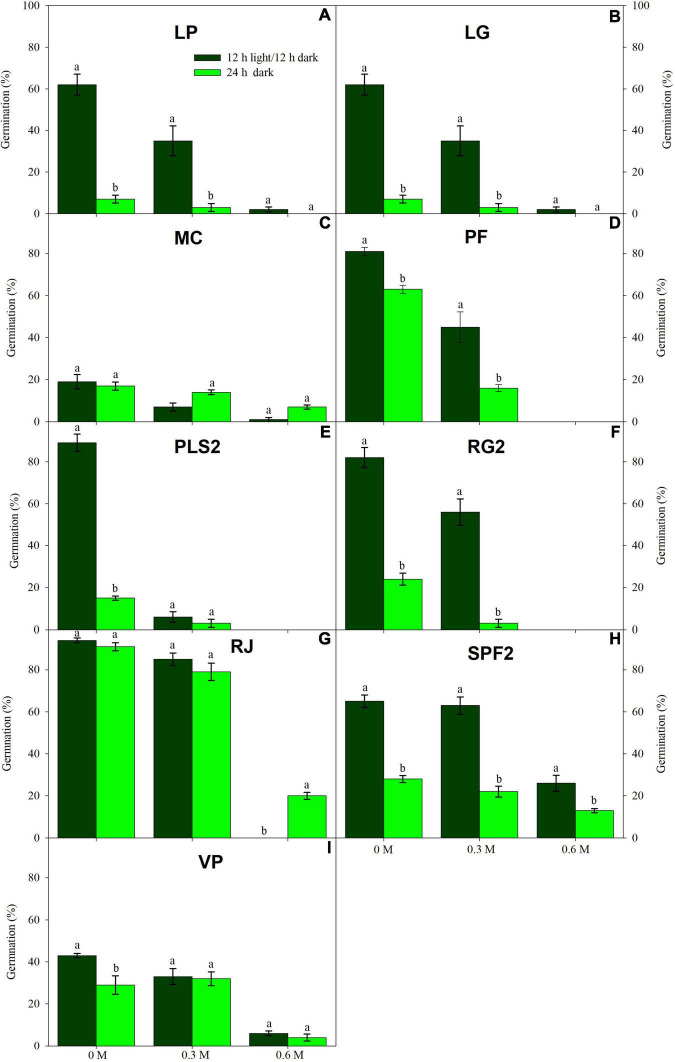
Germination in light (dark green) and dark treatments (light green) under three water PEG6000 concentrations for the remaining nine wetland species occurring in Jiujiang, China. Vertical bars denote ± SE around the mean. Within features, different lowercase letters indicate significance at *P* ≤ 0.05. Vertical bars denote mean (±SE). Within features, different lowercase letters indicate significance at *P* ≤ 0.05. For more details on each study species, see Table 1.

**FIGURE 3:**
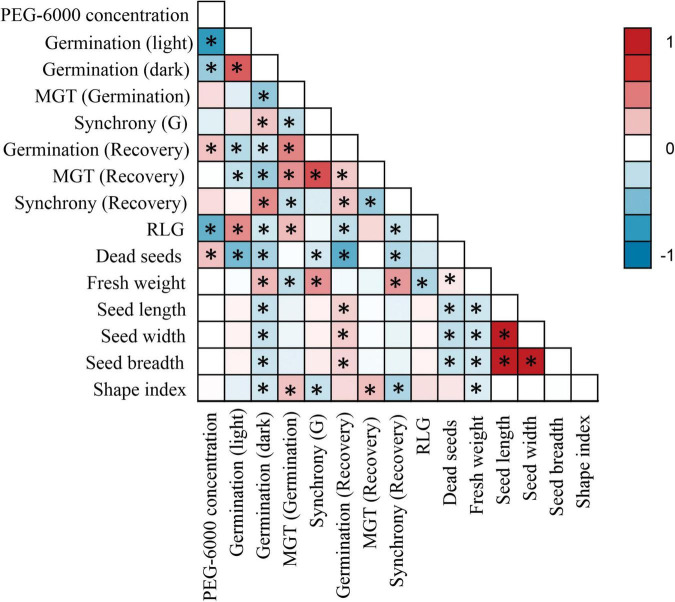
Pairwise correlation matrix between all germination parameters and seed morphology. Asterisks (*) denote a significant correlation of at least *P* < 0.05.

**FIGURE 4:**
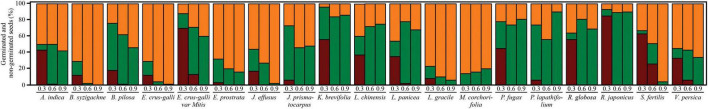
Seeds germinated in PEG (dark red), after stress relief (green), and non-germinated (orange) in each osmotic potential (-MPa) and each plant species. Each bar denotes the mean of four true repetitions.

Low water potentials (i.e., higher PEG6000 concentration) influenced germination percentage (Table 3), with a null germination percent under −0.9 MPa for all study species. Germination of the controls was species-dependent. For instance, seeds of *Melochia corchorifolia* reached only 19.00 ± 3.42%. On the contrary, seeds of *B. pilosa*, *K. brevifolia*, *R. japonicus*, and *R. globosa* showed high G% values of 93, 90, 94, and 82%, respectively. In these species, germination percent was weakly affected by the −0.3 MPa treatment in *R. japonicus* (85.00 ± 2.60%), moderately affected in *K. brevifolia* (56.00 ± 5.89%) and *R. globosa* (56.00 ± 6.32%), and strongly affected in *B. pilosa* (18.00 ± 2.00%) (Figures 1, 2). These data are supported by a direct correlation; as the water potential decreases, the germination percentage decreases with the same intensity, both in light (*r* = 0.742; *P* = 3.75 × 10^–41^) and in dark treatments (*r* = 0.383; *P* = 2.32 × 10^–9^; Figure 3). The MGT varied strongly among the study species, ranging from 2.36 ± 0.25 days in *A. indica* to 25.49 ± 0.17 days in *J. prismatocarpus*. A similar pattern was also demonstrated in seed germination at −0.3 MPa of PEG6000. On the contrary, synchrony variation was very low, ranging from 0.03 ± 0.00 (*P. lapathifolium*) to 0.45 ± 0.50 (*L. chinensis*). The increase of PEG concentration was positively correlated with the proportion of dead seeds (*r* = 0.236; *P* = 3.48 × 10^–3^), while the dead seed was negatively correlated, both in light (*r* = −0.510; *P* = 1.99 × 10^–11^) and dark treatments (*r* = −0.325; *P* = 2.93 × 10^–4^; Figure 4).

**TABLE 3:** Germination, mean germination time, and synchrony of 19 wetland species, occurring in Jiujiang, China. Each value denotes mean (±SE).

Plant species	Germination (%)			Mean germination time (MGT; Days)			Synchrony (Syn)		
	0 MPa	0.3 MPa	0.6 MPa	0 MPa	0.3 MPa	0.6 MPa	0 MPa	0.3 MPa	0.6 MPa
*Aeschynomene indica*	53.00 ± 3.42 a	43.00 ± 5.51 a	1.00 ± 1.00 b	2.36 ± 0.25 a	1.88 ± 0.59 a	2.00 ± 0.71 a	0.12 ± 0.19 b	0.73 ± 0.43 a	–n.d.–
*Beckmannia syzigachne*	23.00 ± 1.91 a	12.00 ± 5.16 b	–n.d.–	7.53 ± 0.35 a	7.50 ± 0.66 a	–n.d.–	0.09 ± 0.07 a	0.04 ± 0.04 a	–n.d.–
*Bidens pilosa*	93.00 ± 1.91 a	18.00 ± 2.00 b	3.00 ± 0.71 c	10.71 ± 0.28 a	11.27 ± 1.15 a	8.50 ± 1.77 b	0.06 ± 0.01 a	0.05 ± 0.03 a	–n.d.–
*Echinochloa crus-galli*	31.00 ± 3.79 a	10.00 ± 1.15 b	–n.d.–	8.67 ± 0.28 b	11.17 ± 0.35 a	–n.d.–	0.05 ± 0.02	–n.d.–	–n.d.–
*Echinochloa crus-galli var. mitis*	75.00 ± 5.51 a	70.00 ± 2.58 a	13.00 ± 4.43 b	5.26 ± 0.28 c	8.59 ± 0.19 b	10.67 ± 1.94 a	0.30 ± 0.03 a	0.08 ± 0.01 a	–n.d.–
*Eclipta prostrata*	37.00 ± 5.74 a	4.00 ± 2.00 b	–n.d.–	10.07 ± 0.58 b	14.50 ± 1.06 a	–n.d.–	0.04 ± 0.02	–n.d.–	–n.d.–
*Juncus effusus*	21.00 ± 1.91 a	17.00 ± 2.52 a	–n.d.–	10.53 ± 0.39 a	11.15 ± 0.22 a	–n.d.–	–n.d.–	0.02 ± 0.02	–n.d.–
*Juncus prismatocarpus*	83.00 ± 3.42 a	6.00 ± 1.15 b	–n.d.–	25.49 ± 0.17 a	23.50 ± 0.50 b	–n.d.–	0.06 ± 0.01 b	1.0 ± 0.01 a	–n.d.–
*Kyllinga brevifolia*	90.00 ± 2.58 a	56.00 ± 5.89 b	–n.d.–	13.33 ± 0.13 b	16.53 ± 0.51 a	–n.d.–	0.09 ± 0.01 a	0.10 ± 0.01 a	–n.d.–
*Leptochloa chinensis*	58.00 ± 2.00 a	37.00 ± 3.42 b	–n.d.–	4.48 ± 0.47 a	5.76 ± 0.28 a	–n.d.–	0.45 ± 0.05 a	0.18 ± 0.04 b	–n.d.–
*Leptochloa panice*	62.00 ± 5.03 a	35.00 ± 7.19 b	2.00 ± 1.15 c	4.39 ± 0.06 c	6.43 ± 0.29 b	19.00 ± 0.01 a	0.32 ± 0.05 a	0.10 ± 0.01 b	–n.d.–
*Lophatherum gracile*	51.00 ± 4.12 a	8.00 ± 2.83 b	–n.d.–	8.49 ± 0.45 b	10.63 ± 0.75 a	–n.d.–	0.06 ± 0.01 a	0.08 ± 0.06 a	–n.d.–
*Melocchia corchorifolia*	19.00 ± 3.42 a	4.00 ± 1.91 b	1.00 ± 1.00 b	8.46 ± 0.96 b	10.21 ± 0.39 b	25.50 ± 0.35 a	0.07 ± 0.07 a	0.17 ± 0.12 a	–n.d.–
*Polypogon fugax*	81.00 ± 1.91 a	45.00 ± 7.37 b	–n.d.–	8.23 ± 0.17 b	12.74 ± 0.92 a	–n.d.–	0.12 ± 0.01 a	0.14 ± 0.04 a	–n.d.–
*Polygonum lapathifolium*	89.00 ± 4.12 a	6.00 ± 2.58 b	–n.d.–	21.85 ± 0.71 a	11.78 ± 3.16 b	–n.d.–	0.03 ± 0.00 b	0.67 ± 0.24 a	–n.d.–
*Rorippa globosa*	82.00 ± 4.76 a	56.00 ± 6.32 b	–n.d.–	6.24 ± 0.27 b	13.00 ± 0.25 a	–n.d.–	0.19 ± 0.02 a	0.06 ± 0.01 b	–n.d.–
*Rumex japonicus*	94.00 ± 1.00 a	85.00 ± 2.6 b	–n.d.–	2.75 ± 0.22 b	3.94 ± 0.14 a	–n.d.–	0.43 ± 0.05 a	0.34 ± 0.01 b	–n.d.–
*Sporobolus fertilis*	65.00 ± 3.00 a	63.00 ± 4.12 b	26.00 ± 3.83 b	5.36 ± 0.36 b	5.53 ± 0.22 b	8.87 ± 0.27 a	0.34 ± 0.04 a	0.24 ± 0.03 b	0.18 ± 0.04 b
*Veronica persica*	43.00 ± 1.00 a	33.00 ± 3.79 b	6.00 ± 1.15 c	5.53 ± 0.57 b	7.03 ± 0.92 ab	9.88 ± 0.31 a	0.10 ± 0.03 a	0.10 ± 0.02 a	–n.d.–

*Within features, different lowercase letters denote significance at P ≤ 0.05.*

### Effects of Seed Traits on Seed Germination

Seed dimensions did not show a significant correlation with germination in the light treatment, but showed a negative correlation with G% in the dark regarding seed length (*r* = −0.216; *P* = 1.04 × 10^–3^), width (*r* = −0.217; *P* = 1.03 × 10^–3^), breadth (*r* = −0.206; *P* = 1.72 × 10^–3^), and shape index (*r* = −0.174; *P* = 8.46 × 10^–3^). There was a positive correlation between dark germination and fresh mass (*r* = 0.273; *P* = 2.93 × 10^–5^).

### Effects of Light on Seed Germination

Light sensitivity of seeds showed a moderate and positive correlation of RLG index with PEG6000 (*r* = 0.578; *P* = 4.15 × 10^–19^). Similarly, RLG positively influenced germination in the light treatment (*r* = 0.438; *P* = 1.03 × 10^–10^) and negatively influenced germination in the dark treatment (*r* = −0.183; *P* = 9.53 × 10^–3^; Figure 4). A positive photoblastism (RLG values close to 1) predominated in at least 13 species (Table 4 and Figures 1, 2). The other three species (*A. indica*, *M. corchorifolia*, and *R. japonicus*) had a negative photoblastism, mostly germinating in dark conditions, with RLG values <0.4 (Table 4). Such negative photoblastism was found at PEG6000 concentration as low as −0.3 or −0.6 MPa. The aphotoblastism (germination indifferent to light, values ∼0.5) was found in *E. crus-galli* var. *mitis*, *L. gracile*, and *V. persica* seeds (Table 4).

**TABLE 4:** Relative light germination (RLG) and probable photoblastism.

Plant species	PEG6000 concentration (M)				Probable
	0	0.3	0.6	RLG_mean_ (±SE)	Photoblastism
*A. indica*	0.51	0.57	0.03	0.37 ± 0.17	Negative
*B. syzigachne*	0.98	1.00	—	0.99 ± 0.01	Positive
*B. pilosa*	0.55	0.34	1.00	0.63 ± 0.20	Positive
*E. crus-galli*	0.81	0.85	—	0.83 ± 0.02	Positive
*E. crus-galli var. mitis*	0.49	0.56	0.37	0.48 ± 0.06	Aphotoblastic
*E. prostrata*	0.98	1.00	—	0.99 ± 0.01	Positive
*J. effusus*	1.00	1.00	—	1.00 ± 0.00	Positive
*J. prismatocarpus*	1.00	1.00	—	1.00 ± 0.00	Positive
*K. brevifolia*	0.99	1.00	—	1.00 ± 0.01	Positive
*L. chinensis*	1.00	1.00	—	1.00 ± 0.00	Positive
*L. panicea*	0.89	0.94	1.00	0.95 ± 0.03	Positive
*L. gracile*	0.74	0.33	—	0.53 ± 0.21	Aphotoblastic
*M. corchorifolia*	0.52	0.32	0.00	0.28 ± 0.19	Negative
*P. fugax*	0.56	0.73	—	0.64 ± 0.08	Positive
*P. lapathifolium*	0.86	0.63	—	0.74 ± 0.12	Positive
*R. globosa*	0.77	0.95	—	0.86 ± 0.09	Positive
*R. japonicus*	0.51	0.52	0.00	0.34 ± 0.17	Negative
*S. fertilis*	0.70	0.74	0.66	0.70 ± 0.02	Positive
*V. persica*	0.60	0.51	0.67	0.59 ± 0.05	Aphotoblastic

*The RLG was calculated as proposed by Flores et al. (2011). All value to RLG denote the media (±SE). More information of each species, see Table 1.*

### Effects of Stress Relief on Seed Germination

Germination strongly increased after stress relief for all study species (Table 5 and Figure 4). Pooling all species responses, the mean germination after −0.3, −0.6, and −0.9 MPa PEG6000 was 32.04 ± 26.94, 39.71 ± 29.70, and 37.02 ± 30.93%, respectively. However, germination patterns strongly varied from species to species after stress relief. Some species, such as *R. japonicus* (77.00 ± 3.70%), *P. lapathifolium* (59.74 ± 1.62%), *B. pilosa* (59.47 ± 2.21%), *L. panice* (58.65 ± 1.01%), *P. fugax* (72.57 ± 0.98%), and *K. brevifolia* (85.08 ± 1.92%), showed G% >55% after stress relief, irrespective of the initial water potential (Figure 4). Seeds of *S. fertilis* (30.85 ± 1.17%), *E. crus-galli* var. *mitis* (45.33 ± 1.00%), *J. prismatocarpus* [(53.00 ± 2.02%) and (30.41 ± 0.90%)], and *A. indica* (34.83 ± 1.15) showed average G% between 30 and 55%. The remaining eight species showed an average G% lower than 30% after stress relief (Figure 4).

**TABLE 5:** Germination, mean germination time, and synchrony of 19 wetland species measured, occurring in Jiujiang, China, after water stress relief.

Plant species	Germination (%)			Mean Germination Time (MGT; Days)			Synchrony (Syn)		
	0.3 MPa	0.6 MPa	0.9 MPa	0.3 MPa	0.6 MPa	0.9 MPa	0.3 MPa	0.6 MPa	0.9 MPa
*Aeschynomene indica*	12.00 ± 2.58 b*	49.58 ± 3.07 a*	42.00 ± 4.76 a	6.00 ± 3.49 a	1.21 ± 0.12 b	0.51 ± 0.39 b	0.33 ± 0.29 b	0.76 ± 0.04 a	0.72 ± 0.05 a
*Beckmannia syzigachne*	5.91 ± 1.55 a*	2.00 ± 1.15 b	–n.d.–	2.25 ± 0.63 b*	8.00 ± 0.71 a	–n.d.–	–n.d.–	–n.d.–	–n.d.–
*Bidens pilosa*	70.91 ± 7.57 a*	61.50 ± 9.22 ab*	46.00 ± 11.94 b	3.63 ± 0.34 b*	5.82 ± 0.27 a	5.84 ± 0.42 a	0.19 ± 0.05 a	0.21 ± 0.06 a	0.09 ± 0.02 b
*Echinochloa crus-galli*	3.00 ± 1.00 a*	4.00 ± 1.63 a	1.00 ± 1.00 a	4.33 ± 0.29 a*	4.67 ± 0.29 a	6.00 ± 0.01	–n.d.–	–n.d.–	–n.d.–
*Echinochloa crus-galli var. mitis*	18.00 ± 2.58 b*	58.00 ± 0.27 a*	60.00 ± 4.32 a	2.68 ± 0.25 b*	4.00 ± 0.27 a*	4.18 ± 0.23 a	0.18 ± 0.05 b	0.20 ± 0.05 b	0.33 ± 0.01 a
*Eclipta prostrata*	29.00 ± 6.61 a	20.00 ± 1.63 a	16.00 ± 1.63 a	8.52 ± 0.43 a	7.07 ± 0.83 a	11.98 ± 2.38 s	0.04 ± 0.02 a	0.04 ± 0.03 a	0.03 ± 0.03 a
*Juncus effusus*	32.52 ± 2.00 a*	27.05 ± 1.09 a	2.00 ± 1.15 b	4.54 ± 0.42 b*	4.72 ± 0.16 b	14.50 ± 1.06 a	0.13 ± 0.03 a*	0.10 ± 0.02 a	–n.d.–
*Juncus prismatocarpus*	67.00 ± 3.42 a*	46.00 ± 6.00 b	46.00 ± 7.39 b	16.60 ± 0.38 a*	16.68 ± 0.18 a	16.65 ± 0.41 a	0.28 ± 0.05 a*	0.04 ± 0.02 b	0.12 ± 0.02 b
*Kyllinga brevifolia*	85.25 ± 7.78 a*	84.00 ± 4.00 a	86.00 ± 5.29 a	6.42 ± 1.23 a*	8.30 ± 0.42 a	6.35 ± 0.19 a	0.09 ± 0.03 b	0.12 ± 0.02 b	0.21 ± 0.03 a
*Leptochloa chinensis*	4.84 ± 1.65 a*	4.00 ± 1.63 a	1.00 ± 1.00 b	4.33 ± 0.29 a*	4.67 ± 0.29 a	6.00 ± 0.01 a	–n.d.–	–n.d.–	–n.d.–
*Leptochloa panice*	30.54 ± 6.44 b	77.42 ± 4.47 a*	68.00 ± 5.89 a	8.91 ± 1.34 a	5.16 ± 0.22 b*	6.06 ± 0.36 b	0.10 ± 0.08 a	0.13 ± 0.01 a	0.18 ± 0.01 a
*Lophatherum gracile*	16.40 ± 1.39 a*	10.00 ± 2.00 b	6.00 ± 1.15 b	5.21 ± 0.16 a*	4.31 ± 0.67 a	6.00 ± 0.58 a	0.13 ± 0.08	–n.d.–	–n.d.–
*Melocchia corchorifolia*	18.22 ± 1.88 a*	14.00 ± 2.58 a*	20.00 ± 1.63 a	5.25 ± 1.12 ab*	2.57 ± 0.68 b*	7.39 ± 1.42 a	0.05 ± 0.03 b	0.23 ± 0.09 a	0.22 ± 0.06 a
*Polypogon fugax*	62.70 ± 7.46 b	74.00 ± 5.77 ab	81.00 ± 5.74 a	5.57 ± 0.46 a	5.52 ± 0.69 a*	6.65 ± 0.91 a	0.12 ± 0.02 a	0.14 ± 0.03 a	0.13 ± 0.02 a
*Polygonum lapathifolium*	70.22 ± 6.08 a*	56.00 ± 2.83 b	53.00 ± 4.43 b	11.62 ± 0.76 b	14.01 ± 0.27 a	9.82 ± 0.50 b	0.06 ± 0.01 a	0.06 ± 0.01 a	0.05 ± 0.01 a
*Rorippa globosa*	3.17 ± 0.39 a*	2.58 ± 0.13 b	3.39 ± 0.43 a	0.73 ± 0.24 a*	1.33 ± 0.14 a	1.84 ± 0.41 a	0.11 ± 0.10 b	0.47 ± 0.02 a	0.34 ± 0.14 a
*Rumex japonicus*	50.00 ± 8.33 b*	91.00 ± 1.66 a	90.00 ± 2.24 a	2.25 ± 0.22 a*	2.23 ± 0.05 a	2.27 ± 0.10	0.75 ± 0.13 a	0.70 ± 0.06 a	0.68 ± 0.08 a
*Sporobolus fertilis*	10.39 ± 3.93 c*	34.10 ± 2.38 b*	48.00 ± 1.63 a	4.50 ± 0.75 a	3.98 ± 0.55 a*	4.36 ± 0.13 a	–n.d.–	0.12 ± 0.06 a	0.15 ± 0.01 a
*Veronica persica*	17.87 ± 5.14 b	39.36 ± 3.65 a*	34.00 ± 5.29 a	4.34 ± 0.28 a*	3.98 ± 0.58 a*	3.92 ± 0.36 a	0.09 ± 0.04 b	0.23 ± 0.02 a	0.15 ± 0.03 ab

Each value denotes mean (±SE). Within features, different lowercase letters denote significance at P ≤ 0.05. Values marked with asterisks (*) are statistically different from those measured durisng water stress.

Germination after stress relief had a weak and inverse but significant correlation with G% in light (*r* = −0.274; *P* = 6.43 × 10^–4^) and dark treatments (*r* = −0.199; *P* = 0.014), showing a direct correlation with MGT under stress (*r* = 0.463; *P* = 2.03 × 10^–5^; Figure 3). For other factors, PEG6000 concentration had a positive correlation (*r* = 0.235; *P* = 3.57 × 10^–3^) influencing germination after stress relief. Similarly, MGT after stress relief showed a negative correlation with seed germination both in light (*r* = −0.228; *P* = 5.74 × 10^–3^) and dark treatments (*r* = −0.372; *P* = 4.02 × 10^–5^), while synchrony had a positive correlation (*r* = 0.435; *P* = 6.23 × 10^–6^) with G% in the dark, and a negative correlation with MGT under stress (*r* = −0.233; *P* = 0.046). Germination after stress relief was negatively correlated with all morphological seed traits, such as seed length (*r* = −0.234; *P* = 0.027), seed width (*r* = −0.239; *P* = 0.019), and seed breadth (*r* = −0.326; *P* = 0.039).

In all study species, embryos unable to germinate after stress relief were subsequently confirmed to be non-viable (dead; Figure 4). The proportion of dead seeds showed a wide variation, between 10% (in *P. lapathifolium* var. *salicifolium* under −0.9 MPa PEG concentration) and 100% (in *B. syzigachne* under −0.9 MPa PEG6000; Figure 4). Germination after stress relief also showed a wide variation between 0% (in *B. syzigachne* under −0.9 MPa PEG6000) and 90% (in *R. japonicus* and *P. lapathifolium*, both initially allowed to germinate at −0.9 MPa PEG6000).

### Germination Traits Grouping and Phylogenetic Analysis

**FIGURE 5:**
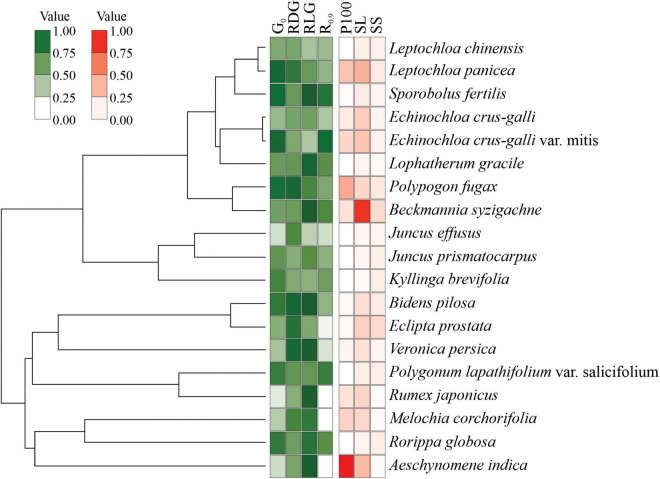
Cluster analysis of species using seed germination parameters.

Cluster analyses indicated three main groups distributed according to germination patterns (Figure 5). Group I is comprised of species with high initial germination in the controls, i.e., non-dormant seeds, but sensitive to drought. Seeds in group II also have no dormancy but are tolerant to drought (germination recovery after stress relief) and tend to be positively photoblastic. Group III comprises seeds with low initial germination and low germination after exposure to the lowest water potential (−0.9 MPa). Such exposure clearly jeopardized G% in these species; for example, seeds of *J. effuses*, *B. syzigachne*, *E. crus-galli*, *E. prostrata*, *L. gracile*, and *M. corchorifolia* could be damaged by drought stress, resulting in non-viable embryos.

**FIGURE 6:**
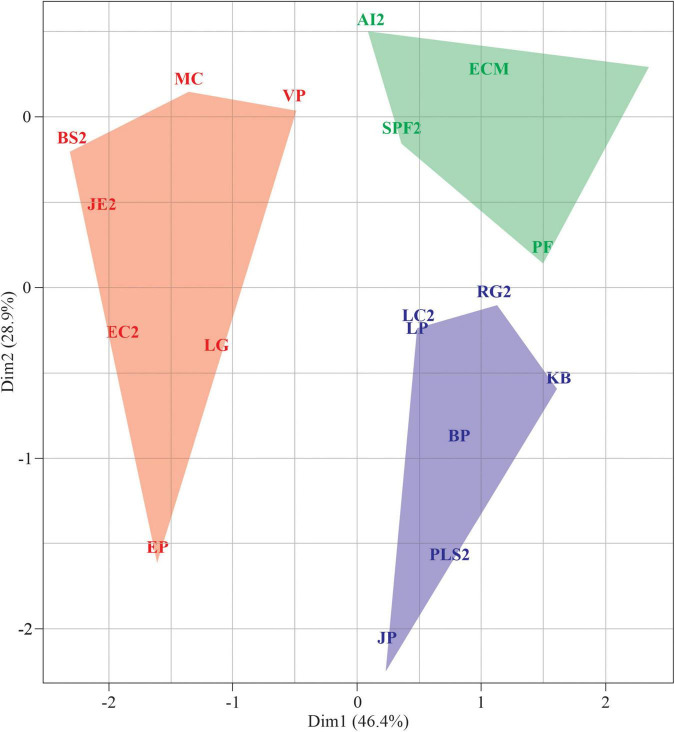
The relation between the plant species phylogenetic relationship and the seed traits. The germination behavior traits were germination with distilled water under white light (G0), relative drought germination (RDG), relative light germination (RLG), and recovery from –0.9 MPa (R0.9). Seed morphology traits were fresh weight of 100 seeds (P100), seed length (SL), and seed shape (SS). The size of the circles corresponds to the magnitude of the mean value inside the measured trait.

No phylogenetic signal has been detected regarding scored values for germination patterns and seed traits (Figure 6). The Pangel’s λ for germination was low (logλ = 4.3, *p* = 1), indicating that the germination parameters had no relation to phylogenetic proximity among the study species. Morphological traits also presented no relation to species’ phylogeny (logλ = 8.45, *P* = 0.29).

## Discussion

The majority of study species were able to germinate at −0.3 MPa PEG6000, decreasing G% at the moderate water potential (−0.6 MPa PEG6000), with little to no germination observed at −0.9 MPa PEG6000. Such results indicate that germination of all study species reached their maximum G% in the controls, while lower water potentials, promoted by PEG6000, decreased germination. Although germination patterns varied greatly from species to species, seedling emergence was unlikely to occur at this level of severe osmotic/drought stress. This life span pattern is commonly attributed to perennial species, since 42% of the species studied (*M. corchorifolia*, *P. fugax*, *R. globosa*, *R. japonicus*, *S. fertilis*, *V. persica*, *A. indica*, and *E. crus-galli* var. mitis) showed significant germination of up to −0.3 MPa and others 21% (*S. fertilis*, *R. japonicus*, *A. indica*, and *E. crus-galli* var. mitis) showed significant germination of up to −0.6 MPa. Germination was more prominent in seeds germinated in light than in darkness with the exception of *M. corchorifolia*. This description is in accordance with Silvertown (1981) and Kooyers (2015), who describe annual species as presenting stress-escaping strategies due to their short life spans, allocating more resources for reproduction in order to maintain their population persistence. However, MGT is a more suitable tool to classify this type of phenomenon. Perennial species tend to have a lower MGT, facilitating rapid seed germination to produce the largest number of plants in the shortest time. In this sense, the species *P. fugax*, *R. globosa*, *R. japonicus*, *V. persica*, and *A. indica* are in synchrony with this theory of life span.

Seeds of a legume shrub commonly used for vegetation recovery in northern China, *Caragana korshinskii*, had germination capacity up to −0.6 MPa, with a subsequent decrease in G% with drought stress (Zheng et al., 2004). Seed germination is the earliest and most sensitive stage in the plant life cycle (Fenner and Thompson, 2005; Baskin and Baskin, 2014). Therefore, germination timing is most likely to synchronize with the favorable season for seedling growth and establishment (Escobar et al., 2018; Bhatt et al., 2020b; Gómez-Maqueo et al., 2020). Temperature may be the most limiting factor for germination and seedling establishment in subtropical monsoon region of China, given that soil moisture availability is not a constraint in the area (Kang et al., 2017). However, due to climate change, droughts are becoming more frequent, and wetland plant communities are likely to be strongly affected through altered habitats (Feng et al., 2016). Understanding the effect of drought stress on the germination of wetland species could be useful for identifying the species that can be tolerant to environmental changes.

Despite the observed germination inhibition under low water potential, seeds of most study species were able to recover their germinability after the alleviation of drought stress. Germination recovery may vary among species. Interspecific differences in germination and recovery responses after alleviating the Ψ_w_ are thus important to understand species’ survival and community assembly under stressful conditions. Such large variation was suggested to be related to a niche differentiation among species to inhabit arid environments, rather than an adaptation (Kos and Poschlod, 2008). On the contrary, the ability to recover after drought has been recognized as an adaptive strategy, selected by the environment to deal with drought stress (Balachowski and Volaire, 2018). We argue that, although germination patterns might be variable, post-stress recovery seems to be a common trait in subtropical wetland communities. Avoiding germination at low water potential and the ability to recover after stress alleviation seems to be a dominant regeneration strategy regarding germination in the study area, as observed in seeds of groups I and II, usually reaching G% >50 after stress relief. Such patterns can help us predict the impact of drought stress and to better understand the adaptative strategies of local plants regarding their regeneration capacity. Moreover, germination recovery was observed even from the lowest water potential, irrespective of phylogenetic constraints, indicating that seeds may undergo some kind of secondary dormancy during drought period and will be able to germinate when water becomes available [see Hegarty (1978)]. In halophyte species from Chinese grasslands, such as *Chloris virgata*, germination recovery reaches up to 80% after stress under severely low water potentials of −1.2 MPa (Lin et al., 2016). Seeds from African savanna trees also show germination requirements related to very stressful conditions, as in the case of *Combretum apiculatum* and *Colophospermum mopane* (Choinski and Tuohy, 1991). Weed grasses, such as *Echinochloa phyllopogon*, and species from desert communities have been reported to show base psi values equal to or lower than −1.0 MPa (Boddy et al., 2012). Intraspecific variations of germination traits have yet to be evaluated for different populations of the study species, as observed for different provenances of *Pinus yunnanensis* and crop wild relatives (Orsenigo et al., 2017; Gao et al., 2021).

Drought stress caused the death of seeds of group III, thus being classified as stress-sensitive species. Different proportions of seed mortality due to drought stress provoked by osmotic solutions (using PEG6000) have been reported in germination studies in the literature (Silva et al., 2001; Miranda et al., 2014; Kintl et al., 2021). For seeds of a tropical legume tree, Silva et al. (2001) described a mean proportion of dead seeds in about 15–18%, probably due to interaction with a germination delay caused by low water potential. This may be due to reduced water potential in orthodox seeds causing embryo death (Chin et al., 1989; Zhang et al., 2021). Moreover, orthodox seeds show a low water potential that is unable to reactivate enzymes to recover germination, leading to embryo death (Garwood and Lighton, 1990; Ebone et al., 2019). In cultivated clover species, the number of dead seeds increased both in the coated (pelleted) seeds and the uncoated ones (Kintl et al., 2021). As expected, we found an enhanced proportion of dead seeds with lower water potentials, mostly in the light treatments.

Seeds germinated in the dark—for instance, buried in soil seed banks—are more likely to recover from water stress than those germinated in light. At the soil surface under full light intensity, seeds are more exposed to water deficit during germination (Taylor and Ewing, 1988). Correlations with seed traits also showed an enhanced proportion of dead seeds with small-seeded species, given that smaller seeds are more likely to suffer injury by drought than the larger/heavier seeds. In spite of the relatively broader tolerance to low water potentials, larger seeds can be supported in drier environments and colonize forest canopy gaps (Bray, 1956). Beckage et al. (2000) showed only slight increases in the recruitment of new seedlings in small gaps, and the increases observed were due to heavier than normal seed production. Clinton (2003) described a greater concentration of small seeds of *Rhododendron maximum* in soils with lower water potential (%Y_w_), while in soils with less negative % Ψ_w_ the presence of larger seeds was significantly greater than small seeds. In agreement with Clinton (2003) and Daws et al. (2008) described a lower risk of seedling establishment as seed size is increased. Conversely, other studies found no correlation between seed mass and drought stress in seeds from the arid Kalahari savanna (Kos and Poschlod, 2008). Further studies should be conducted in other wetland areas worldwide, such as the Brazilian Pantanal, the Congo, and the Nilo Rivers’ basins (see Keddy et al., 2009), to unveil germination patterns and regeneration strategies in threatened floodplains considered as conservation priorities.

## Conclusion

Wetland seeds can be classified into functional groups based on strategies of seed germination and response to drought stress. No phylogenetic signal was found, and thus, environmental factors may influence species’ seed traits, driving regeneration after stress relief. Larger seeds tended to be the most tolerant to drought stress, while the small-seeded species may be injured by the low water potentials. Post-stress germination recovery is a regeneration strategy in subtropical wetland seeds. Seeds of the most study species were able to recover their germinability after the alleviation of drought stress. However, the germination recovery varies among species. Seeds of most of the species were able to recover their germinability from the lowest water potential, irrespective of phylogenetic constraints, indicating that seeds may undergo secondary dormancy during drought, enabling them to germinate when water becomes available.

## Data Availability Statement

The original contributions presented in the study are included in the article/supplementary material, further inquiries can be directed to the corresponding author.

## Author Contributions

AB conceived and designed the experiments. AB and MFP performed the experiments and analyzed the data. AB, DG, AJ-O, and MFP wrote the original draft. LD edited the manuscript and provided editorial advice. All authors approved this submission.

## Conflict of Interest

The authors declare that the research was conducted in the absence of any commercial or financial relationships that could be construed as a potential conflict of interest.

## Publisher’s Note

All claims expressed in this article are solely those of the authors and do not necessarily represent those of their affiliated organizations, or those of the publisher, the editors and the reviewers. Any product that may be evaluated in this article, or claim that may be made by its manufacturer, is not guaranteed or endorsed by the publisher.

## Footnotes

1
http://www.efloras.org/flora_page.aspx?flora_id=2
